# Appropriate fleet selection using dynamic multi-criteria decision making (case study: Gol-E-Gohar Mine No. 3)

**DOI:** 10.1038/s41598-026-46304-4

**Published:** 2026-07-07

**Authors:** Amirhossein Homayounfar, Farhad Samimi Namin

**Affiliations:** 1 Department of Mining Engineering, Amirkabir University of Technology, Tehran, Iran; 2 Department of Mining Engineering, University of Zanjan, Zanjan, Iran

**Keywords:** DMCDM, ANP, Equipment selection, Carbon dioxide emissions reduction, Ecology, Ecology, Engineering, Environmental sciences, Environmental social sciences, Mathematics and computing

## Abstract

The growth of large-scale mining operations presents challenging choices when selecting loading and transport equipment, as these decisions significantly impact output, expenditure, and ecological footprint; poor choices can lead to delays, increased fuel consumption, higher pollution, or damaged equipment. Because mining environments are constantly evolving, decisions must be precise, adaptable, and timely; however, past research has often overlooked the inclusion of time factors and lifecycle phases in fleet planning. This work presents a flexible decision-making method using the Analytic Network Process (ANP) at Gol-E-Gohar Iron Mine No. 3. Its main new feature is that it divides the mine’s lifespan into three periods (start-up, middle phase, and final stage) to track how priorities shift over time. For each phase’s key factors, insights were gathered from 25 mining experts (from universities and industry) through side-by-side evaluations. To enhance consistency while mitigating characteristics or peculiarities associated with phase-only reviews, another Total Mine Life (TML) setup is calculated as a reference point, influencing how technical, financial, and ecological factors are evaluated and assessed at each stage. Instead of relying solely on averages, every ANP comparison, supermatrix run, ranking outcome, and sensitivity check was handled via SuperDecisions software, which also delivered the TML and split-phase outputs used for comparison. Findings show that, compared to fixed methods, the shifting method is more effective or performs better, adjusts more easily, and is more logical or coherent, matching choices to the real-world demands of each interval, so cutting costs and easing ecological strain. In the Gol-E-Gohar No. 3 example, the fleet was first reviewed using the TML benchmark, then across three time blocks, revealing how breaking it into phases helps select suitable equipment or appropriate tools actual conditions at every turn.

## Introduction

### Importance of the problem

Selecting an appropriate loading and haulage fleet is a crucial and impactful decision in open-pit mining operations, as it directly influences operational efficiency, costs, energy use, and environmental impact. An inappropriate fleet setup can result in lower production, more delays, quicker equipment deterioration, higher fuel use, and increased emissions. On the other hand, selecting an optimal fleet is essential for meeting both economic goals and sustainability targets in mining projects. Considering the significant investments involved in loading and haulage systems, errors in fleet choice can cause long-term, often irreversible, effects throughout the entire mine lifecycle^1^.

The problem of haulage fleet selection is inherently multi-criteria, as decision-makers must simultaneously balance technical, economic, and environmental factors that are often conflicting. Under these conditions, multi-criteria decision-making (MCDM) methods are widely used as systematic tools to structure complex decision problems, evaluate criteria, and rank available options. These methods enable the integration of quantitative data with qualitative judgments and expert opinions, making them especially suitable for tackling complex decision-making challenges in mining engineering^2^.

### Literature review

Among MCDM approaches, the Analytic Hierarchy Process (AHP) is one of the most widely used techniques, employing pairwise comparisons within a hierarchical structure to derive criteria weights and rank alternatives, while assuming independence among criteria. To overcome this limitation^3^, the Analytic Network Process (ANP) was developed to explicitly model interdependencies and feedback relationships among criteria and alternatives within a network structure^4^. In contrast, methods such as the Technique for Order Preference by Similarity to Ideal Solution (TOPSIS)^5^ and the VIseKriterijumska Optimizacija I Kompromisno Resenje (VIKOR) are based on measuring the distance of alternatives from positive and negative ideal solutions and are mainly applied to discrete decision problems involving conflicting criteria, with criteria weights typically obtained from complementary methods^6,7^. In addition, to address ambiguity and uncertainty inherent in expert judgments, fuzzy extensions of these methods have been developed and increasingly applied in equipment and system selection problems in mining engineering^8^.

In multi-criteria decision-making models, the decision structure type refers to the manner in which relationships among criteria and alternatives are organized. Hierarchical structures decompose the decision problem into predefined levels and assume independence among criteria and sub-criteria, making them suitable for relatively simple and linear decision problems. In contrast, network structures enable modeling of interdependencies, interactions, and feedback among decision elements and are therefore more effective for complex real-world problems characterized by nonlinear relationships. In some MCDM approaches, decision-making is performed directly based on a decision matrix, in which alternatives are evaluated and ranked according to their performance with respect to the criteria, without explicitly defining a hierarchical or network structure. As summarized in Table 1, different MCDM methods vary in terms of decision structure, modeling capability, and practical limitations^2^.

**Table 1:** Comparison of commonly used multi-criteria decision-making (MCDM) methods in equipment and system selection problems^3,9,10^.

MCDM method	Decision structure type	Consideration of interdependencies	Advantages	Limitations
AHP	Hierarchical	No	Simple and transparent structure; ease of implementation; suitable for well-structured problems	Assumes independence among criteria; unable to model complex interactions
ANP	Network-based	Yes	Ability to model interdependencies and feedback among criteria; suitable for complex real-world problems	Higher computational complexity; requires careful expert judgment
TOPSIS	Decision matrix	No	Computational simplicity; intuitive interpretation; wide applicability	Does not derive criteria weights internally; ignores relationships among criteria
VIKOR	Decision matrix	No	Effective for conflicting criteria; focuses on compromise solutions	Highly dependent on externally determined criteria weights
Fuzzy AHP	Hierarchical	No	Improved handling of linguistic judgments and uncertainty compared to classical AHP	Increased complexity; still assumes independence among criteria
Fuzzy ANP	Network-based	Yes	Combines modeling of interdependencies with uncertainty representation; suitable for complex decision environments	Computationally intensive; time-consuming decision process
Fuzzy TOPSIS	Decision matrix	No	Effective ranking under uncertainty; relatively simple implementation	Does not capture interactions among criteria; relies on external weights
Hybrid MCDM	Hybrid	Depends on model	High flexibility; simultaneous mitigation of weaknesses of individual methods	Strong dependence on model design; reduced transparency in some cases

To provide an overview and facilitate comparison of commonly used MCDM approaches in the literature, Table 1 summarizes key characteristics of these methods regarding decision structure type, ability to account for interdependencies among criteria, and their main advantages and limitations. This comparison emphasizes that the suitability of an MCDM method depends on the nature of the decision problem, the complexity of relationships among criteria, and the level of realism needed in the decision model, thus offering a basis for justifying the choice of the proposed approach in this study. Given the importance of the problem and the effectiveness of MCDM approaches in supporting complex decision-making, a substantial body of research has focused on the selection of loading and haulage fleets in mining operations. The following section reviews the most significant studies in this area.

Accordingly, loading and haulage fleet selection has attracted extensive attention in the literature, and a variety of optimization and decision-making methods have been proposed to address the associated challenges, An early and widely cited example is Basçetin (2004)^11^, who employed the AHP to enhance group decision-making and reduce the time required for equipment selection.Bazzazi et al.^12^ proposed a hybrid AHP–TOPSIS model under fuzzy conditions to manage uncertainty and imprecise data in fleet selection. This approach was further developed by Aghajani et al.^13^, who introduced a fuzzy MCDM framework for optimizing drilling, loading, and hauling equipment combinations. Around the same period,Campanella & Ribeiro^14^ introduced a foundational framework for Dynamic MCDM, incorporating time-sequenced evaluations for more adaptive decision-making. Additionally, Subtil et al.^15^ developed a multi-stage fleet dispatch system to enhance productivity and reduce costs. May^16^ followed with the application of queuing theory to improve truck-shovel haulage systems, demonstrating its effectiveness in optimizing fleet utilization and lowering transportation costs.Rahimi Ghazikalayeh et al.^17^ combined FANP and TOPSIS to assess equipment performance under complex decision-making conditions. De Sousa et al.^18^ applied MCDM to select trucks for a bauxite mining company, achieving cost reductions and productivity improvements. Salama^19^ employed simulation and multiple linear regression to predict haulage requirements during mine closure stages, with a focus on cost efficiency. Benlaajili et al.^20^ developed a two-stage optimization framework that combines simulation and linear programming to improve fleet allocation. As attention to sustainability grew, Patyk et al.^21^ introduced a comprehensive methodology that integrated environmental and ore quality criteria with technical and economic factors. In the same year, Mohtasham et al.^22^ employed a mixed-integer nonlinear programming (MINLP) model to optimize the truck fleet size, resulting in significant cost savings and productivity gains. A wave of studies in 2022 further advanced the field.Huerta et al.^23^ utilized simulation and the firefly metaheuristic algorithm to optimize fleet performance, reporting increased productivity and reduced idle time. Musbah et al.^24^ applied the Imperialist Competitive Algorithm for truck allocation, resulting in improvements in ore production and waste reduction.. Mohtasham et al.^25^ introduced a real-time decision-making framework to support fleet sizing and dispatching in open-pit mines Anaraki & Afrapoli^26^ presented a bi-objective model that balanced environmental and economic considerations, highlighting the role of truck speed and age in fleet efficiency.Ccatamayo-Barrios et al.^27^ compared AHP and TOPSIS for mining method selection, concluding that AHP outperforms TOPSIS in extraction evaluations. Francis & Thomas^28^ developed a hybrid system dynamics–MCDM model for sustainability assessments with emphasis on renewable energy integration.Samimi Namin et al.^29^ introduced a comprehensive methodology for selecting mine transportation systems, employing the analytic hierarchy process alongside FUZZY-TOPSIS, specifically for large-scale copper mining. Most recently, Moradi et al.^30^ proposed a nested fleet management system (N-FMS) that improved fleet capacity by 14.6%, Čelebić et al.^31^ proposed a fuzzy logic-based model to support decision-making under uncertain mining conditions. In parallel,Dimitrijević et al.^32^ introduced a hybrid fuzzy MCDM model to select optimal land reclamation methods in open-pit mines, identifying afforestation as the most effective strategy. Although numerous studies have addressed the selection of loading and haulage fleets in mining operations, most have relied on static decision-making models that assume constant criterion priorities and lack the ability to capture temporal changes or interactions among criteria over time. Samimi Namin et al.^33^ applied a grey MCDM model to compare diesel and trolley-assisted trucks in an Iranian open-pit mine and found that trolley-assist can lower haulage costs and emissions. The study demonstrates that grey MCDM is useful for evaluating low-carbon fleets under uncertainty.

### Research gap

Although previous studies have significantly contributed to developing decision-support tools for haulage fleet selection, a key review of the literature shows that most current methods depend on static decision-making models. These studies assume that criteria weights and decision-maker preferences stay constant over time, and fleet selection is usually done for a single point in time or operational scenario.

However, real mining operations exhibit a dynamic, phase-dependent nature, in which factors such as mining depth, haulage distance, production rate, cost structure, and environmental constraints evolve over the mine life cycle. Neglecting these temporal changes limits the ability of static models to capture shifts in criteria priorities and the long-term implications of fleet selection decisions. As a result, a substantial gap exists between many proposed models in the literature and the actual operational conditions encountered in mining practice. This limitation persists even in some of the most recent studies that employ newly developed decision-making techniques. For instance, Samimi Namin et al.^33^ and Amou et al.^34^ applied the relatively new SECA method to evaluate alternative equipment configurations in mining applications. Despite methodological advancements, these studies still relied on static evaluations and did not explicitly account for temporal non-stationarity or phase-dependent changes over the mine life cycle. This observation indicates that the neglect of time-dependent decision dynamics remains an unresolved issue in the current literature, even when modern MCDM approaches are employed.This analysis makes it clear that the addressed problem goes beyond a single case study and reflects a broader challenge in phase-dependent decision-making for complex engineering systems.

### Objectives and contributions of this study

To address this gap, the present study proposes a Dynamic Multi-Criteria Decision-Making (DMCDM) framework based on the Analytic Network Process. The proposed approach enables the systematic revision and updating of criteria weights across the different stages of the mine life cycle. In this framework, the mine life cycle is divided into distinct time phases, and the decision structure is defined in a stage-oriented manner, allowing technical, economic, and environmental priorities to be evaluated separately for each phase.

The key contribution of this framework lies in its ability to maintain overall decision consistency across the total mine life while explicitly capturing gradual changes in operational conditions and decision-maker preferences. Consequently, the fleet selection process is transformed from a static, one-time decision into a flexible, adaptive decision-support tool that more accurately reflects real mining conditions and supports strategic planning across different project stages.

The main contributions of this study can be summarized as follows:

- Development of a dynamic ANP-based MCDM framework for loading and haulage fleet selection in open-pit mining operations.
- Explicit modeling of changes in technical, economic, and environmental criteria priorities across different stages of the mine life cycle.
- Introduction of a phase-based comparison strategy alongside a total mine life reference scenario to enhance decision consistency.
- Demonstration of the applicability of the proposed framework through a real-world case study at Gol-E-Gohar Iron Mine No. 3.

The remainder of this paper is structured as follows. Section Methodology introduces the proposed decision-making framework and research methodology. Section Results details the case study and how the framework was applied at Gol-E-Gohar Iron Mine No. 3. The results and analysis are presented in Section Discussion, followed by discussion, limitations, and concluding remarks in Section Conclusion.

## Methodology

The methodology section is structured into two main subsections. The first subsection Proposed framework presents the proposed decision-making framework, outlining its theoretical foundations, analytical structure, and computational procedure. The second subsection Case study (Gol-E-Gohar Mine No. 3) focuses on the case study, where the proposed framework is applied step by step using real operational data to demonstrate its practical implementation and validity.

### Proposed framework

#### Theoretical background of dynamic MCDM

The Dynamic Multi-Criteria Decision Making (DMCDM) method is specifically designed for real-world problems where evaluations are carried out sequentially over different periods, and previous assessments impact future decisions. The primary feature of this approach is its ability to update criteria and alternatives over time, leveraging historical data to enhance decision-making. The concept of DMCDM is outlined in Eqs. 1 to 5 as presented by Campanella and Ribeiro^14^.

represents a set of time moments, which may be infinite. $A_t$ and $C_t$ denote the set of alternatives and criteria, respectively, available at time $t \in T$. For each $C_t$, a corresponding weight vector is defined.

1 $$\begin{aligned} W_t \in [0,1]^n \quad \text {and} \quad \sum _{w \in W_t} w = 1 \quad \text {for} \quad t \in T. \end{aligned}$$

To link historical evaluations with current assessments, it is necessary to define a historical set. The purpose of this set is to retain alternatives that may influence the next phase of decision-making. The historical set of alternatives is defined as follows:

2 $$\begin{aligned} H_0 = \varnothing , \qquad H_t \subseteq \bigcup _{t' \le t} A_{t'} \quad \text {for} \quad t \in T. \end{aligned}$$

The main process of Dynamic Multi-Criteria Decision Making (DMCDM) is described as follows. A static ranking for each alternative is obtained using the classical Multi-Criteria Decision Making (MCDM) method, which is represented by:

3 $$\begin{aligned} R_t : A_t \rightarrow [0,1]. \end{aligned}$$

In the second stage, at each time $t \in T$, the final ranking of each alternative is determined using an evaluation function:

4 $$\begin{aligned} E_t : A_t \cup H_{t-1} \rightarrow [0,1], \qquad t \in T. \end{aligned}$$

which is defined as:

5 $$\begin{aligned} E_t(a) = {\left\{ \begin{array}{ll} R_t(a), &  a \in A_t \setminus H_{t-1}, \\ D_E\big (E_{t-1}(a),\, R_t(a)\big ), &  a \in A_t \cap H_{t-1}, \\ E_{t-1}(a), &  a \in H_{t-1} \setminus A_t. \end{array}\right. } \end{aligned}$$

The $D_E$ operator is a fully reinforcing aggregation operator used to combine past and present evaluations. It is important to note that the aggregation operators used in static and dynamic ranking are entirely independent.

Dynamic multi-criteria decision-making (DMCDM) offers several advantages, making it highly suitable for complex and real-world problems. This method provides high flexibility in adjusting criteria and alternatives over time, allowing it to adapt to dynamic environments and changing conditions. Additionally, DMCDM can capture relationships between sequential evaluations across different time periods, leading to more optimal decision-making. By utilizing aggregation operators and feedback mechanisms, it effectively integrates both historical and current information, enhancing the accuracy and stability of the results. The ability to incorporate past data while maintaining ongoing evaluations makes dynamic multi-criteria decision-making a powerful tool for applications such as supplier selection, project management, and decision-making under uncertainty Tao et al.^35^.

The theoretical formulation of Dynamic Multi-Criteria Decision Making(DMCDM) provides a general foundation for incorporating temporal dependencies into decision-making problems. However, the effectiveness of a DMCDM approach in real-world applications depends on selecting an appropriate multi-criteria evaluation method that models complex relationships among decision criteria. In this study, the Analytic Network Process is adopted as the core decision-making mechanism within the DMCDM framework, due to its ability to capture interdependencies and feedback among criteria and alternatives. The integration of ANP with a dynamic decision structure enables a coherent and flexible framework for evaluating loading and haulage fleet alternatives across different stages of the mine life cycle.

#### ANP-based decision model

The Analytic Network Process (ANP) is an advanced multi-criteria decision-making method developed by Saaty as a generalization of the AHP to handle complex decision problems involving interdependencies and feedback among decision elements. While AHP is based on a strictly hierarchical structure and assumes independence among criteria and alternatives, ANP overcomes this limitation by replacing the hierarchy with a network structure, allowing the modeling of both internal and external dependencies among decision elements. This feature is essential within the DMCDM framework because real-world mining problems often involve structural interdependencies and changes in decision priorities over time^36^.

**Fig. 1:**
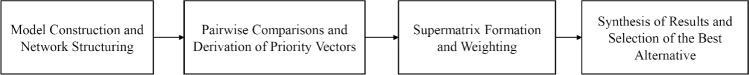
Main procedural steps of the ANP^37^.

**Table 2:** Pairwise comparison scale used in ANP (Saaty’s 1–9 scale)^36^.

Numerical value	Description	
	Preference statement	Interpretation
9	Extremely preferred	Absolute importance
7	Very strongly preferred	Very strong significance
5	Strongly preferred	Strong significance
3	Moderately preferred	Medium importance
1	Equally preferred	Equivalent importance

Intermediate values: 2, 4, 6, 8 (Medium values).

As shown in Fig. 1, the ANP-based decision-making process follows a structured four-step procedure, providing a clear framework for modeling interdependencies, deriving priorities, and selecting the most appropriate alternative^37^.

- *Step 1: Model Construction and Network Structuring* In ANP, the decision problem is modeled as a network composed of clusters (components) and elements. Each cluster $C_k$ consists of $n_k$ elements denoted as $e_{k1}, e_{k2}, \ldots , e_{kn_k}$, and influence relationships may exist among elements within the same cluster or between different clusters. These relationships, which show direct or indirect influences among decision elements, form the basis of the ANP network structure. In a DMCDM context, this network can be revisited at various decision stages or lifecycle phases to explicitly reflect changes in operational conditions and shifts in criteria priorities over time. The ANP procedure begins by structuring the decision model and identifying relationships among clusters and elements, typically based on expert knowledge, prior studies, or supporting analytical methods.
- *Step 2: Pairwise Comparisons and Derivation of Priority Vectors* In the next step, pairwise comparisons are conducted to evaluate the relative influence of elements on one another. These comparisons are performed using Saaty’s fundamental 1–9 scale, where a value of 1 indicates equal importance and higher values represent increasing degrees of preference or influence. Table 2 presents the numerical scale used for pairwise comparisons in ANP. Within the DMCDM framework, these pairwise comparisons are carried out in a stage-wise manner to capture the evolution of decision-makers’ preferences over time^38^. To ensure the reliability of expert judgments, the consistency ratio (CR) is calculated for each pairwise comparison matrix. Pairwise comparisons with a CR value less than 0.1 are considered acceptable. The local priority vectors derived from these comparisons constitute the primary inputs for constructing the ANP supermatrix at each decision stage.
- *Step 3: Supermatrix Formation and Limiting Process* The supermatrix is a block matrix that represents all relationships among clusters and elements within the decision network. Each submatrix $W_{ij}$ represents the influence of elements in cluster *i* on those in cluster *j*, with its columns corresponding to the local priority vectors derived from the relevant pairwise comparisons. When there is no relationship between two clusters, the corresponding submatrix is zero^4^. Based on the derived local priority vectors, the ANP supermatrix is constructed to capture the interdependencies among decision elements, as represented in Eq. 6.
  6 $$ {\text{w = }}\left( {\begin{array}{*{20}c}    {C_{1} } &  \cdots  & {C_{k} } &  \cdots  & {C_{N} }  \\    {W_{{11}} } &  \cdots  & {W_{{1k}} } &  \cdots  & {W_{{1N}} }  \\     \vdots  &  \ddots  &  \vdots  &  \ddots  &  \vdots   \\    {W_{{k1}} } &  \cdots  & {W_{{kk}} } &  \cdots  & {W_{{kN}} }  \\     \vdots  &  \ddots  &  \vdots  &  \ddots  &  \vdots   \\    {W_{{N1}} } &  \cdots  & {W_{{NK}} } &  \cdots  & {W_{{NN}} }  \\   \end{array} } \right) $$
  Since the columns of the initial supermatrix may not meet the column-stochastic property, the supermatrix is transformed into a weighted supermatrix. During this process, the cluster weights–derived from pairwise comparisons regarding the overall goal–are applied to the corresponding submatrices, ensuring each column in the weighted supermatrix sums to one. This column-stochastic property is essential for the supermatrix to converge. By raising the weighted supermatrix to successive powers, the direct and indirect influences among elements are disseminated throughout the network, and the matrix reaches a stable state called the limit supermatrix. This limit supermatrix reflects the long-term influence of each element on all others in the network and provides the final priorities of the decision elements. When the supermatrix represents the entire decision network, the final priorities can be directly read from the columns of the limit supermatrix^39^.
- *Step 4: Synthesis of Results and Selection of the Best Alternative* In this study, ANP is used as the main computational method for weighting and ranking criteria and alternatives within the proposed DMCDM framework. The integration of the network-based structure of ANP with a dynamic decision-making approach allows for the consideration of structural interdependencies among criteria and changes in priorities over different stages of the mine life cycle.

#### Overview of the proposed DMCDM–ANP framework

In most prior studies on loading and haulage fleet selection, decision-making has been based on static approaches, and temporal changes in mining operating conditions across the mine life cycle have not been explicitly incorporated into the decision model. However, factors such as extraction depth, haulage distance, cost structure, environmental requirements, and managerial priorities evolve, and neglecting these changes may lead to decisions that are poorly aligned with the mine’s actual operating conditions.

To address this limitation, this study proposes a dynamic multi-criteria decision-making framework that models fleet selection as a stage-wise decision process over the mine life cycle. Within this framework, the temporal dimension of decision-making is incorporated through the Dynamic Multi-Criteria Decision Making (DMCDM) approach, which updates criteria weights and alternative rankings across stages.

The computational core of the proposed framework is based on the ANP. ANP is selected because the fleet selection problem exhibits a network-based structure in which decision criteria may mutually influence one another. Unlike hierarchical methods and many ranking-based approaches that assume independence among criteria, ANP explicitly models interdependencies and feedback relationships among criteria and alternatives, thereby providing a more realistic representation of the decision structure.

**Fig. 2:**
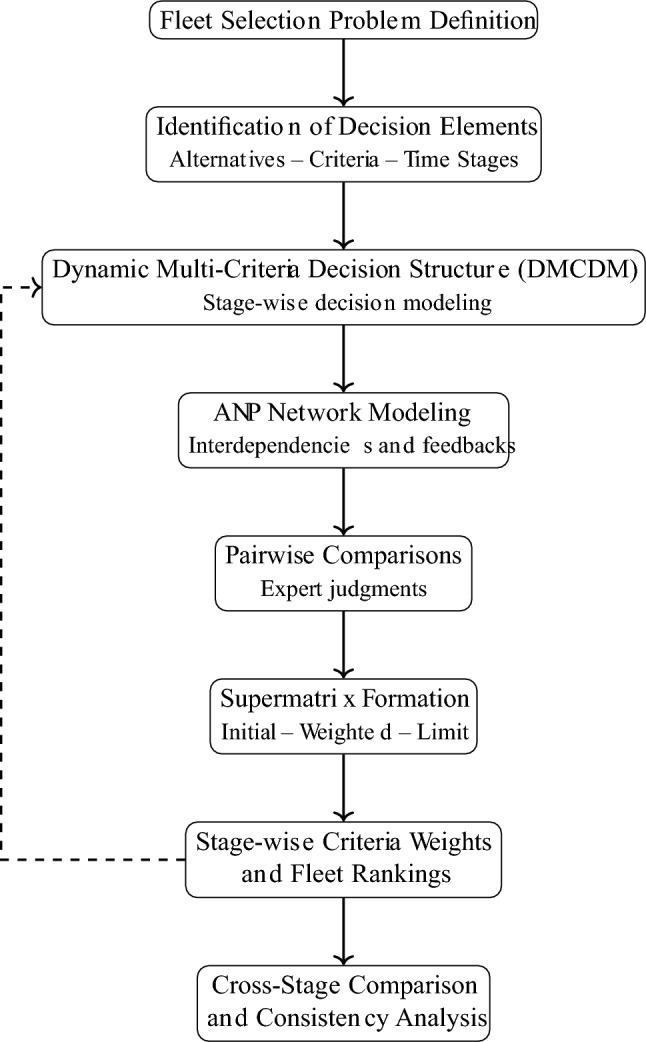
Conceptual workflow of the proposed DMCDM–ANP framework for dynamic fleet selection.

Overall, the proposed framework consists of several main steps. First, the decision alternatives, evaluation criteria, and time stages are defined. Then, for each time stage, the decision network and dependency relationships among criteria and alternatives are established, and pairwise comparisons are performed. Based on these comparisons, ANP supermatrices are constructed, and stage-specific criteria weights and alternative rankings are derived. Finally, the stage-wise results are analyzed and compared in a consistent manner. The operational sequence of these steps is illustrated schematically in Fig. 2,which presents the conceptual workflow of the proposed DMCDM–ANP framework developed in this study. As shown in this workflow, the fleet selection problem is first defined, followed by the identification of decision elements, including alternatives, evaluation criteria, and time stages. The decision process is then structured dynamically using a stage-wise DMCDM approach to explicitly account for temporal variation across the mine life cycle. For each time stage, an ANP-based network model is constructed to capture interdependencies and feedback relationships among decision criteria and alternatives.

#### Time-based structuring of mine lifecycle

**Fig. 3:**
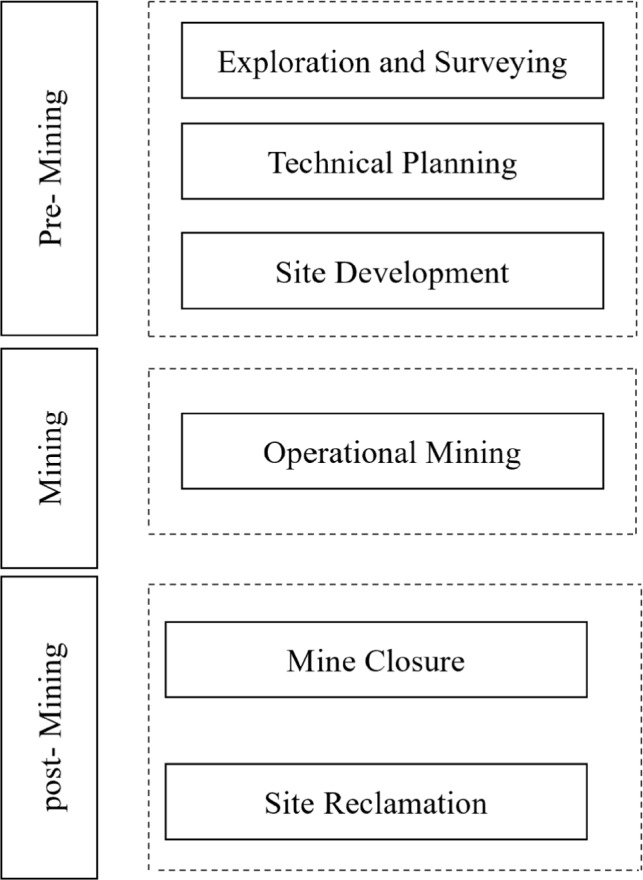
Modified mine life-cycle stages used for temporal segmentation.

To effectively incorporate the time dimension into decision-making, it is crucial to divide the mine’s lifecycle into separate operational phases. This division not only improves the structure and clarity of the evaluation framework but also makes sure that all decision-making criteria— including economic, operational, and environmental aspects— are meaningfully captured across different stage. Since priorities, constraints, and operating conditions change throughout the mine’s lifecycle, a stage-based approach significantly enhances the relevance and strength of multi-criteria analyses. Prior research has emphasized the importance of such structuring. For instance, Tolvanen et al.^40^ and Dorukan et al.^41^ Both advocate dividing the mine lifecycle into three main phases for sustainability evaluations and operational planning: (1) Pre-mining, (2) Mining, and (3) Post-mining. This division matches real-world mining workflows and supports flexible decision-making suited to each stage’s specific needs, especially in fleet selection, investment decisions, and sustainable resource management. Figure 3 provides a clear visualization of the modified mine life-cycle stages used for temporal segmentation in this study. As shown, the entire mining lifecycle is divided into three main phases: pre-mining, active mining, and post-mining. Each phase includes a series of activities such as exploration, planning, operation, and reclamation, reflecting the practical and regulatory progression of mining processes. This segmentation allows for a more realistic and phase-specific analysis of fleet selection priorities throughout the entire mine life.

- *Pre-mining Phase*- This phase covers exploration, mine planning, and infrastructure setup before operations begin. Activities generally include geological surveys, reserve assessments, engineering design, and obtaining key permits such as exploration and initial environmental approvals. Major decisions about project viability and funding are made mainly during this phase.
- *Mining Phase*- This stage involves active mineral extraction and operational production. During this phase, the mine operates at or near full capacity, focusing on cost efficiency, safety, and productivity. Operational permits are maintained, and upgrades or expansions may be pursued. Criteria such as operational expenditures and production performance are central to decision-making during this stage.
- *Post-mining Phase*- As mineral resources are depleted, the focus shifts to environmental restoration and closure planning. This phase involves site cleanup, infrastructure shutdown, soil stabilization, and ecological rehabilitation. Regulatory compliance, corporate social responsibility, and environmental sustainability guide decision-making priorities, while remaining costs and risk mitigation measures shape final actions.
- *Total mine life(TML)*- To improve understanding of how decision outcomes evolve over time, a benchmark evaluation is also carried out by treating the fleet selection problem as a single decision stage across the entire mine lifespan. This comprehensive assessment serves as a reference point, enabling clearer comparisons between stage-specific results and the overall baseline.

Therefore, the temporal intervals analyzed in this study are as follows:

- *Total mine life(TML)* – considered as the reference scenario;
- *Pre-mining stage* – encompassing early-stage planning and development activities;
- *mining stage* – representing the period of full-scale ore extraction;
- *Post-mining stage* – covering closure and site reclamation efforts.

#### Criteria selection

To ensure comprehensive coverage of the different aspects of decision-making, this study’s framework is based on three main categories of criteria: economic, technical-operational, and environmental. These three dimensions serve as the core pillars of analysis and together address key factors such as cost, operational efficiency, and environmental impact when evaluating alternatives. Since each domain includes a wide range of influential indicators and parameters, the final criteria selection was guided by two complementary methods: first, their frequency of use in previous research; and second, references to the latest articles published within each specialized area. As a result, four criteria were chosen: capital cost (initial investment), operational cost (ongoing expenses), equipment capacity, and carbon dioxide emissions. This combination ensures a balanced consideration of different decision-making factors and aligns with current literature in mining and multi-criteria decision-making studies. Additionally, recent scientific research confirms that these four criteria remain widely used, reliable, and relevant in the latest mining-related publications for evaluating performance and choosing extraction equipment, highlighting their ongoing importance in both research and industry.

- *Economic Criteria*- In the economic analysis of mining projects, effectively combining capital and operational costs captures all key economic aspects of the project^42^. Together, these two factors serve as vital economic indicators, offering a comprehensive view of expenses, revenues, and financial risks. Capital cost, which is the initial investment, significantly impacts equipment choices and project financing strategies. Meanwhile, operational costs, reflecting ongoing expenses like operation, maintenance, and repairs during the equipment’s lifespan, directly influence economic efficiency and the useful life of the equipment. These economic factors are also key components in the decision-making model proposed by aghajani et al.^13^, whose study emphasizes the importance of economic impacts when selecting mining equipment. The alignment of goals and needs between this study and their model supports the logical reasoning for selecting these economic criteria in the current project’s decision-making framework.
- *Technical Criterion*- In this study, capacity is defined as a critical technical criterion for evaluating the loading and hauling abilities of equipment. This factor is vital for choosing machinery that can efficiently move large volumes of material while maximizing productivity and minimizing resource use. Although economic and environmental analyses address different aspects of a mining project, using inaccurate or unsuitable technical criteria can greatly diminish the practical value of the evaluation. Therefore, carefully selecting precise technical indicators is crucial for obtaining meaningful and reliable results. It is noted that technical parameters such as safety and reliability are also frequently emphasized in the existing literature as important criteria in mining equipment selection. However, due to the lack of reliable, consistent, and case-specific data for these parameters in the present study, they could not be explicitly incorporated into the decision-making model. Consequently, equipment capacity was adopted as a representative technical criterion, as it implicitly reflects several key operational aspects associated with safety, reliability, and overall technical performance under real operating conditions. Capacity specifically impacts important operational factors such as machinery wear, maintenance and repair costs, operator productivity, time management, and fuel efficiency–underscoring its importance in assessing technical performance. The significance of this criterion has been acknowledged in multiple studies. For example, Bazzazi et al.^12^ utilized capacity to evaluate equipment performance in terms of haulage efficiency and operational effectiveness. Likewise, Mohtasham et al.^25^ and Patyk et al.^21^ highlighted capacity as a critical technical factor in fleet selection, further confirming its vital role in performance assessments.
- *Environmental Criterion*- Alongside economic and technical factors, environmental impacts have become increasingly crucial in evaluating and selecting loading and hauling equipment. With climate change emerging as a global challenge, industrial decision-making must now systematically account for carbon footprints and long-term environmental effects. This issue becomes even more significant in mining projects because of the heavy reliance on fossil fuels by mining machinery. As noted by soon et al.^43^ and shahbakhsh et al.^44^, carbon dioxide is the dominant human-made greenhouse gas, significantly contributing to climate change. It accounts for the largest share of global warming and, unlike many other pollutants, has a long atmospheric longevity, accumulating and causing long-term climate effects. Therefore, emission levels have become a decisive indicator in assessing the environmental impacts of industrial equipment. In this study, emissions are calculated using an emission factor of 2.68 kilograms of carbon dioxide per liter of diesel fuel burned. This specific factor offers a clear and dependable basis for measuring the carbon footprint of different equipment options, helping to identify less polluting alternatives. As a result, the $CO_2$ emission criterion not only highlights the research’s focus on environmental sustainability but also acts as a practical tool to align mining operations with sustainable development goals, thereby minimizing harmful impacts on the climate and ecosystems.

#### Criteria weighting

**Fig. 4:**
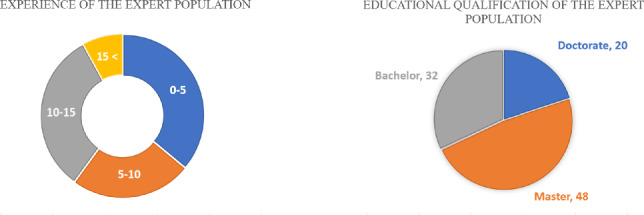
Information of the statistical society of experts.

The weighting of criteria in this study was conducted across the defined time intervals of the mine’s lifespan to accurately capture variations in decision-making priorities at each stage. To determine the weight of each criterion, expert opinions were gathered using an Expert System approach (Fig. 4). In this process, 25 specialists active in mining engineering were consulted. These experts were divided into two main groups: the first included academic professionals (such as university faculty members and graduate students at the master’s or doctoral level), while the second consisted of industry practitioners. Overall, 60% of participants were academics, and the remaining 40% were industry professionals. Regarding educational background, 20% of the experts held a doctorate, 48% held a master’s degree, and 32% held a bachelor’s degree. Additionally, the professional experience of the expert panel showed a balanced distribution: 36% had 0–5 years of experience, 24% had 5–10 years, 32% had 10–15 years, and 8% had over 15 years of experience in mining-related activities. A significant portion of the respondents (about 40%) also had direct experience in selecting mining machinery or had worked as mining engineers at Gol-E-Gohar Mine No. 3, which further increased the reliability of the results and ensured closer alignment with actual operational conditions.

**Fig. 5:**
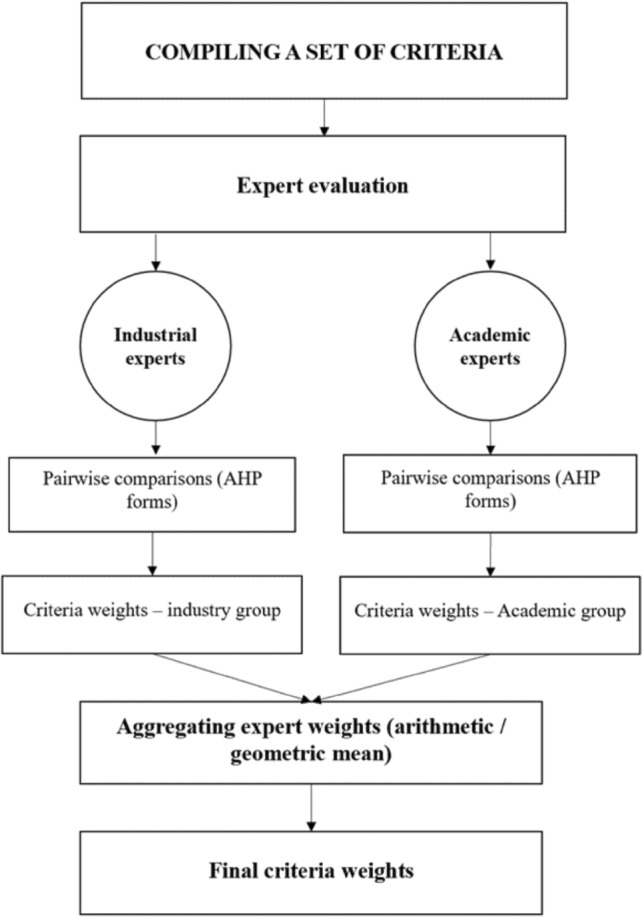
Research framework for aggregating expert opinions and ranking mining alternatives.

According to Fig. 5, a pairwise comparison matrix of the criteria was created and distributed to all experts to evaluate the relative importance of each criterion compared to the others in each time period. After collecting the surveys, the data were averaged to determine the final, combined weights reflecting the collective judgment experts.

**Fig. 6:**
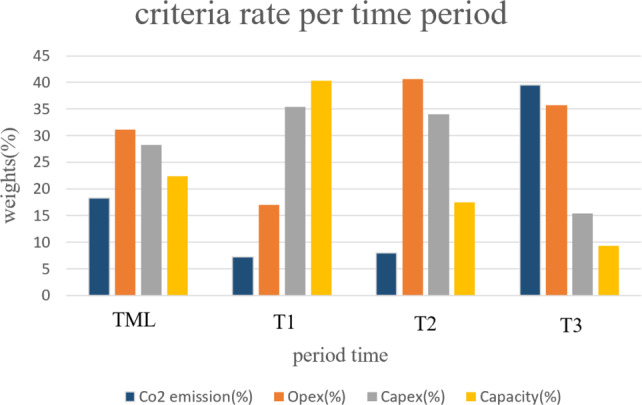
The different values of each criterion in each time period.

These aggregated weights were later input into the SuperDecisions software and incorporated within the ANP–DMCDM framework to facilitate the decision-making process for selecting the optimal loading and haulage fleet. Consequently, the expert-based weighting method, by including a variety of academic and industrial viewpoints, allowed the development of a valid, realistic, and operation-focused set of criterion weights that accurately represent the conditions at each stage of the mine’s lifecycle. Figure ?? and Table 3 show how the weights of each main criterion change across the three defined periods of the mine’s life and for its entire duration (TML). They also demonstrate how decision-makers’ priorities shift from the initial to the middle and finally to the end-of-mine stage. These values are direct outputs from the SuperDecisions software, which, after inputting the expert-derived pairwise comparison matrices, built and normalized the supermatrix (Fig. 6). In this process, the criteria weights were first entered, then the fleet scenarios were added to the software. Next, the entries related to the criteria cluster in the weighted supermatrix were extracted and systematically reported in the table. Therefore, the numbers in Table 1 are not just simple averages of expert opinions; rather, they are weights that reflect both the internal interdependencies among the criteria in the ANP model and the temporal distinctions ($T_1$, $T_2$, $T_3$, and TML). As a result, they form the quantitative basis for decision-making regarding the selection of loading and haulage fleets during each period.

**Table 3:** Criteria weights across mine life stages (TML and phases 1–3).

Time	Capacity (%)	Capex (%)	Opex (%)	CO_2_ emission (%)	Total (%)
TML	22.345	28.274	31.134	18.245	100
1	40.286	35.438	16.990	7.286	100
2	17.399	33.965	40.685	7.951	100
3	9.359	15.419	35.726	39.497	100

Table 4 presents the results of the pairwise comparisons among the four main decision-making criteria–namely Capacity, Capital Expenditure (Capex), Operational Expenditure (Opex), and carbon dioxide emissions ($CO_2$ emission)–across different stages of the mine life cycle, including the total mine life (TML) and the three time intervals corresponding to the beginning (T1), middle (T2), and end (T3) of the mine life. The values reported in the table are determined solely based on expert judgments in the fields of mining engineering, engineering economics, and environmental studies. These judgments were collected through pairwise comparison questionnaires distributed among 25 experts, comprising a combination of academic researchers and industry practitioners, and were aggregated using average values to reflect the collective perspective of the expert panel for each time period.

**Table 4:** Pairwise comparison values of criteria across mine life-cycle stages.

Compared criteria	TML	T1	T2	T3
Capacity vs. Capex	1/2	1/2	1/2	1/2
Capacity vs. CO$_2$	1/5	1/5	1/3	1/5
Capacity vs. Opex	1/3	1/3	1/3	1/5
Capex vs. CO$_2$	3	4	3	3
Capex vs. Opex	3	2	2	3
CO$_2$ vs. Opex	2	1/3	1/5	1/2

As observed, the relative importance of the criteria changes significantly throughout the mine’s life cycle, reflecting shifts in managerial priorities and project goals at different stages. During the early phase (T1), the Capacity criterion has the highest weight among the criteria, indicating a strong focus on maximizing production and efficient resource use at the beginning of mining operations. At this stage, quickly reaching production targets and recovering the initial investment are crucial. In contrast, during the final phase of the mine’s life (T3), the importance of Capacity drops considerably, while environmental criteria–especially carbon dioxide ($CO_2$) emissions–become much more significant. This shift shows an increased focus on environmental sustainability, regulatory compliance, and reducing environmental impacts as mining progresses. Additionally, comparing values across all time periods reveals that Capital Expenditure (Capex) consistently remains a higher priority than Capacity (with a pairwise comparison ratio of 2:1), emphasizing the ongoing and decisive role of economic factors throughout the project and in choosing loading and haulage equipment. These trends highlight that decision-making for mining equipment selection requires an understanding of how priorities evolve over time and cannot depend on a single fixed criterion across all stages.

Overall, these values are used as inputs to the decision-making model to determine the weight of each criterion for each time period. The expert-based weighting method, by integrating diverse perspectives from both academic and industry communities, offers a credible, realistic, and operationally aligned foundation for decision-making in selecting loading and haulage equipment.

#### Ethics approval and consent to participate

In this study, the judgments of a panel of 25 experts were used to determine the weights of the criteria for the multi-criteria decision-making analysis. The study was conducted through an online questionnaire involving experts affiliated with the University of Zanjan and Amirkabir University of Technology, as well as mining industry professionals. Prior to participation, the purpose of the research and study procedures were explained to all experts, and participation was entirely voluntary. Written informed consent was obtained from all participants, who were free to withdraw from the study at any stage.

No personally identifiable or sensitive personal information was collected, and all responses were analyzed in aggregate form. As this research was based on expert judgment collected through a voluntary questionnaire and did not involve medical experiments or sensitive personal data, formal ethical approval was not required according to institutional research procedures. All methods were carried out in accordance with relevant guidelines and regulations.

### Case study (Gol-E-Gohar Mine No. 3)

**Fig. 7:**
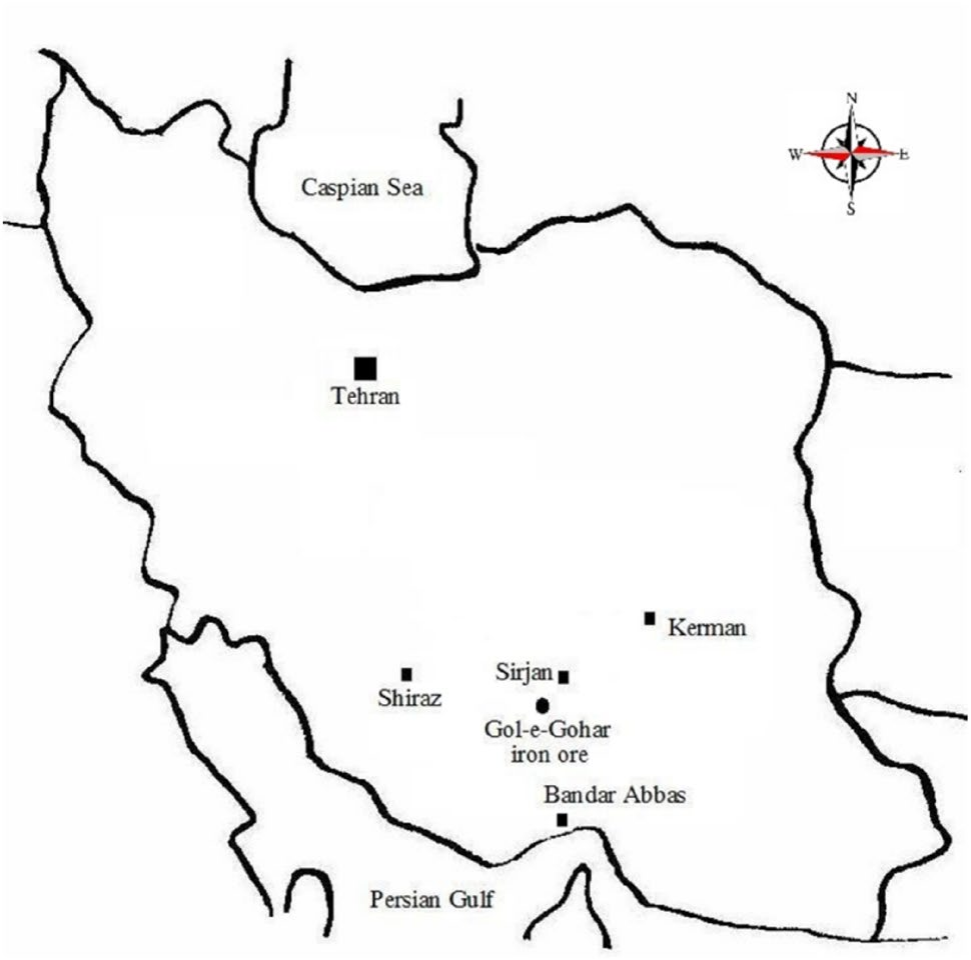
Schematic map showing the approximate location of Gol-E-Gohar Mine No. 3 in Iran. The map was manually created by the authors using Microsoft Paint (Microsoft Corporation, Version 11, https://www.microsoft.com) for illustrative purposes only.

The Gol-E-Gohar Iron Ore Mine No. 3 is amongst the most important mining locations in Iran and is run by Gohar Zamin Company. It lies in Kerman Province, on the northern flank of the Sanandaj-Sirjan metamorphic zone, and is approximately 55 km southwest of Sirjan, meaning it is centrally located within Iran’s mining network; Fig. 7 displays this. Access to the location is good, with major industrial areas connecting directly via the Sirjan-Shiraz highway. Additionally, mineral shipments will have the advantage of the nearby Bafgh-Bandar Abbas double-track rail line, located just 8 km to the east. Furthermore, a private rail link connects Gol-E-Gohar to the wider Iranian railway system, ensuring that iron ore moves smoothly and at lower costs within the nation and beyond its borders.

This robust infrastructure enables the mine to play a significant role in Iran’s steel and mining sectors, thereby boosting economic development in the region. Its strategic location ensures smooth connections with transportation routes, speeding up ore shipments and increasing efficiency. These benefits strengthen the country’s industrial supply chains and help expand Iran’s presence in international markets panahi et al. (2014)^45^. The Gol-E-Gohar mining region, characterized by an arid and desert climate, is situated on the alluvial plains of the northern foothills of the Zagros Mountains. The area experiences cold, dry winters, hot, dry summers, and mild springs with limited rainfall. The average annual precipitation is 65.5 mm, with most rain falling in winter and spring; spring rains are often accompanied by flash floods. The region’s morphology consists of a vast plain with carbonate outcrops and an average elevation of 1,736 meters, surrounded by northwest-southeast trending outcrops reaching elevations of 1,966 meters.

#### Equipment selection scenarios

The definition and selection of the four scenarios examined in this study were based on available technical–economic data and documentation. Specifically, the initial information regarding the type and number of loading and haulage equipment was obtained from the technical–economic report prepared by ADC, the mine design consultant for Gol-E-Gohar Mine No. 3. In this report, the fleet configuration was described in a static manner, with a fixed number of machines assigned to each stage of the mining operation.

However, since the main goal of this study is to explore decision-making behavior within a dynamic and time-sensitive framework, the static fleet configuration mentioned earlier was used only as an initial baseline and was then refined using an engineering-based approach. In this context, drawing on classical fleet design and loading–haulage balance principles discussed in the mining engineering literature–particularly those in Hartman’s textbook^46^ and the authors’ operational experience at Gol-E-Gohar Mine No. 1, which is close to Gol-E-Gohar Mine No. 3 and has a similar reserve size, several key technical factors were included in the scenario definition process.

For instance, maintaining a balance between the capacity of loading equipment and the number of haul trucks, as a key principle of fleet design in open-pit mines, played an important role in shaping the scenarios of this study. Accordingly, the ratio of haul trucks to shovels and loaders in the proposed scenarios was chosen to prevent excessive waiting times for loading equipment or idle haul trucks, while also avoiding operational bottlenecks within the haulage system. These considerations are clearly reflected in the differences between high-capacity scenarios (like Scenario B) and those with moderate or lower capacities (such as Scenarios C and D), ensuring that any increase in the number of haul trucks is consistent with the loading capacity. This approach helps prevent reductions in overall system efficiency, which has been identified in mining engineering literature as a common mistake in fleet design.

Additionally, the practical feasibility of the scenarios under real mine conditions was seen as a key constraint. This means that the scenarios had to be not only theoretically sound but also compatible with existing infrastructure, mine road widths, maintenance capacity, and workforce availability at the case-study mine. As a result, fleet configurations that would require unrealistic increases in equipment or major changes to mine infrastructure were excluded from the selection process.

Finally, the scenarios were designed to encompass a variety of production capacity levels, capital expenditures, and operational costs to facilitate the analysis of decision-making behavior under different conditions and stages of the mine’s life cycle. This intentional diversity ensures that the chosen scenarios are representative of commonly used and practically feasible fleet configurations in large open-pit mining operations.

It should be noted that although, from a theoretical perspective, many other equipment combinations could have been identified, these options were excluded from the analysis due to their incompatibility with the mine’s actual operational conditions. Therefore, the final scenarios were chosen as a representative and engineering-focused set capable of reflecting realistic and implementable decision-making options within the mine’s operational environment.

**Table 5:** Initial decision matrix for ANP.

Scenario	Equipment	Number	Capacity (ton)	Capex (M$)	Opex (M$/month)	CO_2_ emission (kg/Day)
A	Shovel (15 m^3^)	3				
	Loader (10 m^3^)	3	2115	441	22	3658
	Haul truck (126 ton)	15				
B	Shovel (15 m^3^)	4				
	Loader (10 m^3^)	4	3475	672	35	5664
	Haul truck (126 ton)	25				
C	Shovel (15 m^3^)	3				
	Loader (10 m^3^)	2	1697	339	18	2908
	Haul truck (126 ton)	12				
D	Shovel (15 m^3^)	2				
	Loader (10 m^3^)	1	1400	273	14	2248
	Haul truck (126 ton)	10				

Table 5 shows the raw performance data of the four fleet scenarios (A–D) and serves as the initial decision matrix in this study. Each scenario details the types and numbers of equipment used (Shovel 15 m³, Loader 10 m³, Haul Truck 126 tons). For each scenario, four main indicators are calculated: Capacity (tons), Capex (million USD), Opex (million USD/month), and $CO_2$ emissions (kg/day). Capacity is based on the nominal productivity of the equipment, explicitly considering availability and utilization factors; Capex indicates the capital costs for procurement and installation; Opex includes the recurring monthly expenses for fuel, maintenance, and manpower; and $CO_2$ emissions are estimated using fuel-specific emission factors based on consumption. These data are used as the initial inputs for the ANP method in each time period ($T_1$–$T_3$). In later steps, the values are normalized and combined with weights from ANP pairwise comparisons to build the weighted supermatrix and ultimately determine the final ranking of the scenarios. The Capacity criterion is benefit-oriented (higher values are better), while Capex, Opex, and $CO_2$ are cost/risk-oriented criteria (lower values are better).

**Fig. 8:**
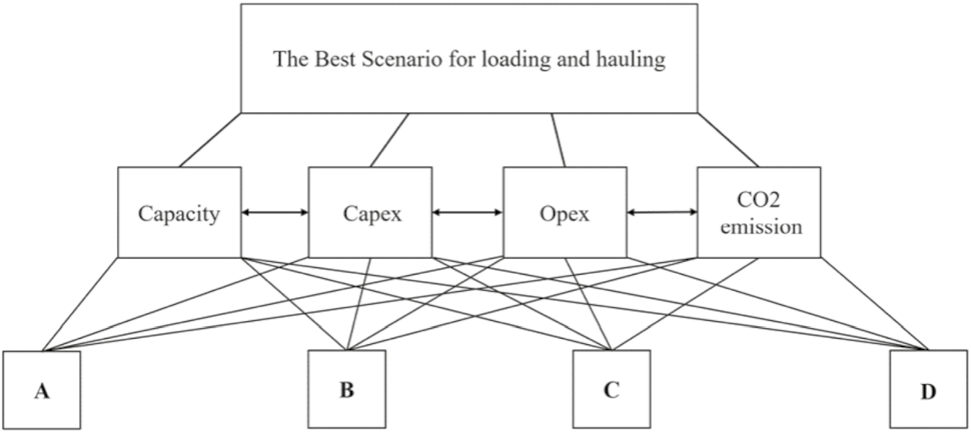
Criteria and scenarios for selecting the best equipment scenario.

Figure 8 illustrates the decision-making process for selecting the best scenario for loading and hauling equipment. The four alternatives (A, B, C, D) are assessed based on four main criteria: Capacity, Capital Expenditure (Capex), Operational Expenditure (Opex), and CO2 Emissions. The arrows depict the relationships and influences between the criteria and the alternatives, aiming to find the most efficient and sustainable mining choice operations.

### Weighted supermatrix

The analysis of the weighted supermatrix results reveals a meaningful shift in criterion priorities over time. In the first phase (T1), production capacity held the highest importance (40.2%), reflecting a strong focus on achieving initial production targets. In the second phase (T2), operational costs became the top priority (40.6%), indicating an emphasis on economic efficiency during the mine’s stable production period. Finally, in the third phase (T3), $CO_2$ emissions gained the greatest weight (39.5%), highlighting the increasing importance of environmental considerations in the mine’s end-of-life stage. This progression demonstrates that the adopted decision-making model effectively captures the dynamic and realistic evolution of priorities throughout the mine’s lifecycle (Table 6). The flexibility of the weighted supermatrix in capturing complex dependencies and considering changes over time makes it a highly effective tool for multi-criteria decision-making. It empowers decision-makers to make more precise, informed, and well-rounded choices, taking into account the evolving needs of the mine. Overall, the use of the weighted supermatrix has been pivotal in providing clarity to the decision-making process and suggesting the most optimal solutions.

**Table 6:** Weighted supermatrix problem for each time period.

Period	Senario	Capacity	Capex	Opex	CO_2_ emissions
TML	A	0.20450	0.11750	0.11750	0.11750
	B	0.58612	0.05529	0.05529	0.05529
	C	0.10957	0.26220	0.26220	0.26220
	D	0.09981	0.56501	0.56501	0.56501
$T_1$	A	0.37363	0.19271	0.18007	0.17595
	B	0.43570	0.08007	0.04973	0.05908
	C	0.13491	0.27072	0.29179	0.28857
	D	0.05577	0.45650	0.47841	0.47619
$T_2$	A	0.2655	0.2622	0.2622	0.2622
	B	0.57073	0.56501	0.56501	0.56501
	C	0.0984	0.1175	0.1175	0.1175
	D	0.6537	0.5529	0.5529	0.5529
$T_3$	A	0.26650	0.07007	0.11750	0.11750
	B	0.57703	0.10226	0.05529	0.05529
	C	0.09840	0.26986	0.2622	0.2622
	D	0.06537	0.55782	0.56501	0.56501

## Results

### Baseline analysis over the total mine life (TML)

**Fig. 9:**
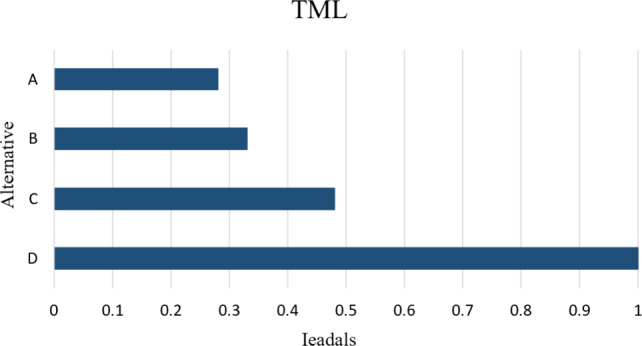
Normalized final scores of fleet alternatives in the baseline (Total Mine Life) scenario.

To establish a consistent reference for phase-wise comparisons, the final scores of the fleet alternatives were initially calculated and normalized over the TML scenario. In this step, technical, economic, and environmental criteria were combined under fixed macro-assumptions and assessed within the ANP–MCDM framework using the SuperDecisions software. Figure 9 presents the normalized final scores of the alternatives on a [0,1] scale and shows their baseline ranking. Under the TML condition, Scenario D achieves the highest normalized score, followed by Scenarios C, B, and A. These results provide the baseline against which phase-specific outcomes in subsequent time periods are compared.

### Early stage of mine life

**Fig. 10:**
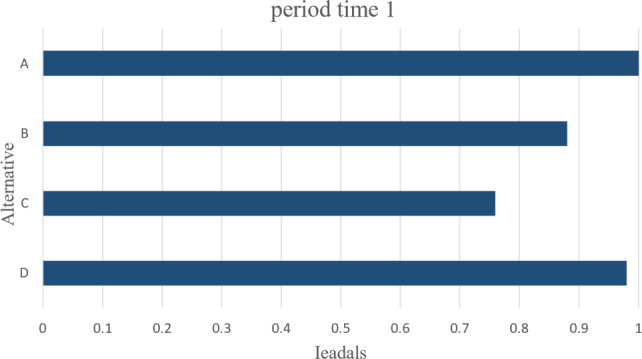
The degree of closeness of each scenario to the ideal in the first time period.

Figure 10 shows the normalized closeness of each fleet scenario to the ideal solution during the early stage of mine life ($T_1$). During this period, Scenario A achieves the highest score (1.000) and ranks first. Scenarios D and B follow closely, as the second and third options, respectively, while Scenario C ranks fourth. The small differences among the top scenarios’ scores indicate competitive performance levels among the alternatives at this stage.

### Mid-stage of mine life

**Fig. 11:**
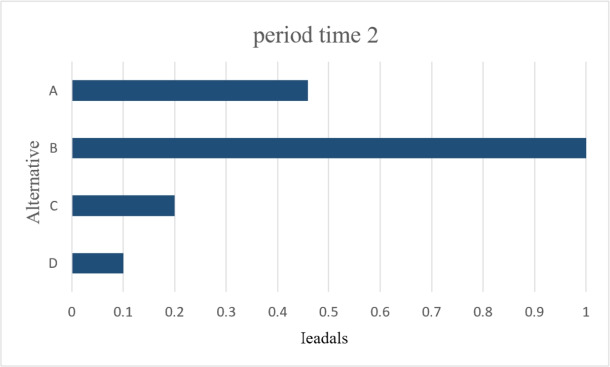
The degree of closeness of each scenario to the ideal in the second time period.

The results for the mid-life stage of the mine ($T_2$) are illustrated in Fig. 11. During this period, Scenario B achieves the highest normalized score (1.000) and clearly ranks first among the alternatives. Scenario A is in second place, followed by Scenarios C and D in third and fourth, respectively. The significant gap between Scenario B and the other choices highlights its superior overall performance under the weighting structure established for this stage.

### End of mine life

**Fig. 12:**
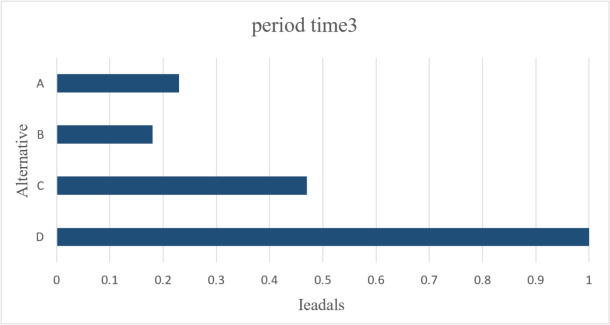
The degree of closeness of each scenario to the ideal in the third time period.

Figure 12 presents the results for the end-of-life stage of the mine ($T_3$). In this period, Scenario D achieves the highest normalized score (1.000) and is ranked first. Scenario C follows as the second-ranked option, while Scenarios A and B are ranked third and fourth, respectively. The ranking pattern observed in this stage differs notably from those of earlier periods.

### Integrated evaluation of temporal priorities throughout the mine lifecycle

**Fig. 13:**
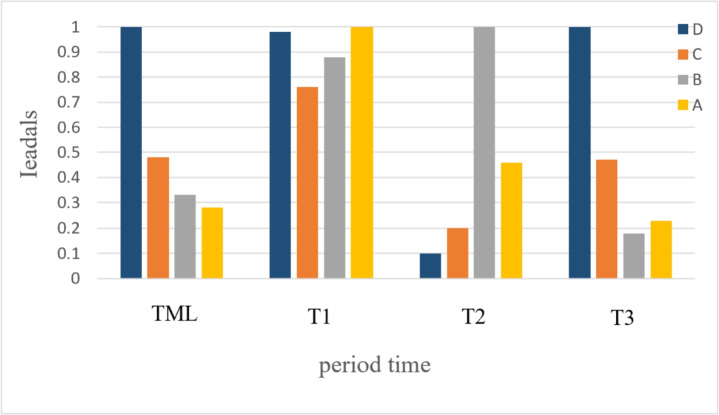
The degree of closeness of each scenario to the ideal state in each time period.

Figure 13 summarizes the normalized rankings of all fleet scenarios under the TML condition and across the three time periods ($T_1$–$T_3$). Under the TML baseline, Scenario D attains the highest score, followed by Scenarios C, B, and A. In the early stage ($T_1$), Scenario A ranks first, whereas Scenario B becomes dominant during the mid-life stage ($T_2$). In the final stage ($T_3$), Scenario D regains the top position. This sequence highlights the variation in scenario rankings across different phases of the mine life cycle.

### ANP sensitivity

**Fig. 14:**
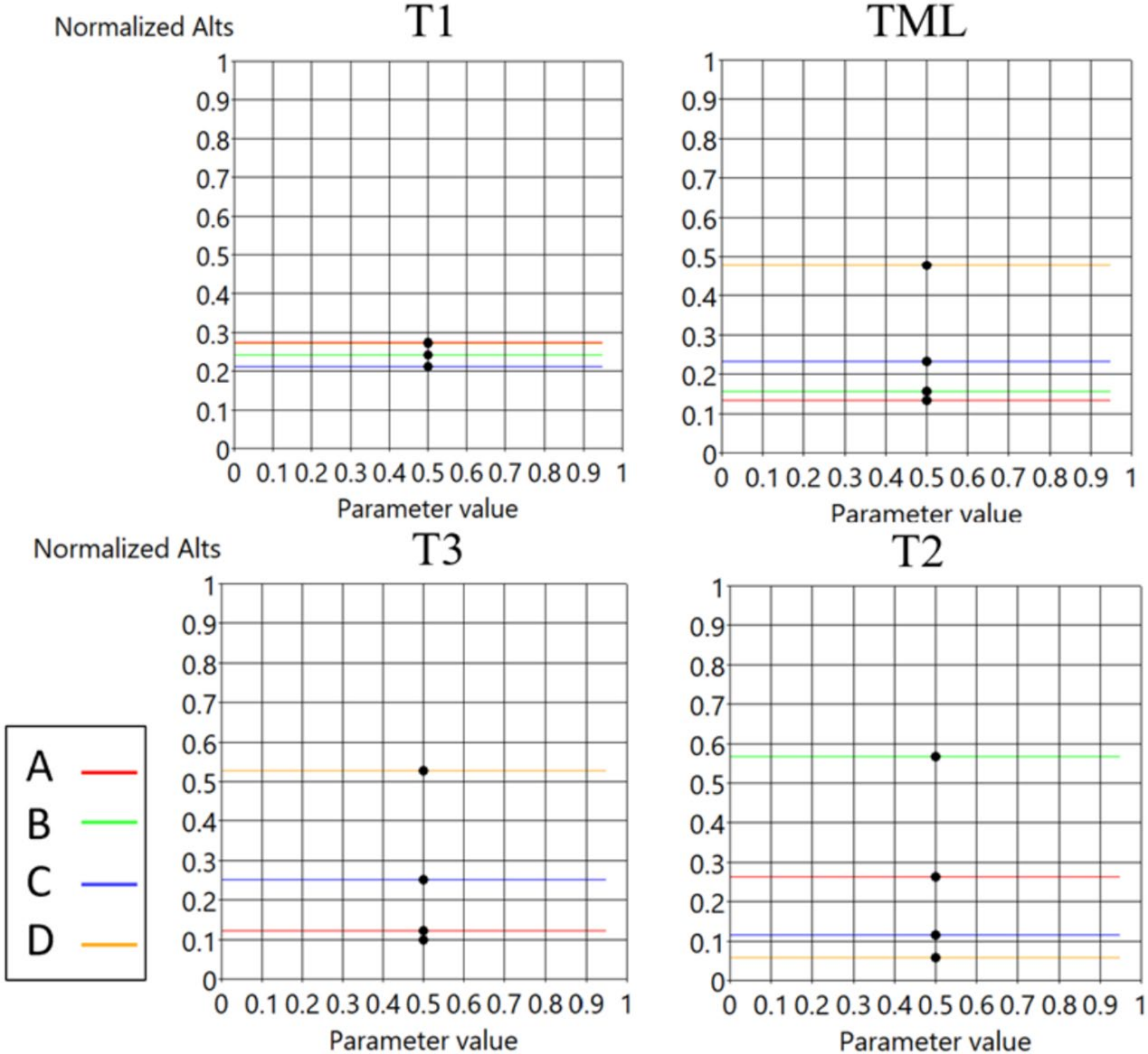
Phase-wise ANP sensitivity (SuperDecisions).

Figure 14 presents the ANP sensitivity analysis results for the TML, $T_1$, $T_2$, and $T_3$ scenarios. The sensitivity charts were generated using the Sensitivity Analysis module of the SuperDecisions software. In each panel, the horizontal axis represents variations in the control parameter value within the [0,1] range, while the vertical axis shows the normalized priorities of the fleet alternatives.

#### TML

Under the TML condition, Scenario D has the highest normalized priority across the entire parameter range, followed by Scenarios B, C, and A. The absence of line intersections indicates a stable ranking structure as parameters change.

#### Early stage ($T_1$)

In the $T_1$ sensitivity panel, the normalized priority lines of all four scenarios are closely spaced, indicating similar performance levels. Scenario A maintains a slight advantage over the other alternatives throughout the parameter range.

#### Mid-life stage ($T_2$)

During the $T_2$ stage, Scenario B remains clearly dominant across the entire parameter range, with no rank reversals observed. The gap between Scenario B and the other scenarios shows a stable ranking outcome.

#### End-of-life stage ($T_3$)

In the $T_3$ sensitivity panel, Scenario D consistently has the highest priority value, followed by Scenario C. The stability of this ranking across parameter variations confirms the robustness of the results at this stage.

## Discussion

To compare dynamic and static approaches in selecting loading and haulage fleets, it is enough to note that static models assume constant criterion weights throughout the entire analysis period. Under this assumption, information from different stages of the mine life cycle is inevitably combined into a single decision framework. While this simplification facilitates computation easier, it may lead to decisions that do not fully match the actual operational conditions of specific mine phases, especially in projects with characteristics that are inherently time-dependent.

In the proposed framework, the ANP was used as the core of the decision-making model to explicitly consider interdependencies and feedback among technical, economic, and environmental criteria. Unlike simple hierarchical structures, ANP allows modeling interactions such as the link between operating costs and equipment availability or the relationship between fuel use and carbon dioxide emissions–interactions whose strength and importance change throughout the mine’s life cycle. Combining this with dividing the mine’s life into three distinct stages (T1, T2, and T3) enabled adjustment of criterion weights and their effects based on each phase’s actual conditions.

The results from this approach show that evaluating based on Total Mine Life (TML) gives only a broad, average view of scenario performance, while phase-specific analysis reveals important shifts in decision priorities over time. Specifically, in the early stage of mine life (T1), Scenario A performs better because of the higher emphasis on production capacity and the decision-makers’ focus on rapidly achieving production targets. As the operation moves into the mid-life stage (T2), where mining activities stabilize and controlling costs becomes more important, Scenario B becomes the leading choice because of its better operational costs and economic efficiency. In the final stage (T3), with growing environmental concerns and the decreasing importance of production capacity, Scenario D ranks highest due to its better performance in reducing carbon dioxide emissions and its alignment with end-of-mine requirements. These results clearly show that choosing the right fleet is not a one-time, static decision but a dynamic process that needs ongoing reassessment as mine conditions change over time.

From a managerial perspective, these findings highlight key practical implications. The results indicate that fleet replacement or renewal timing should not be based solely on initial project conditions but should be adjusted according to the mine’s transition from one stage to the next. A fleet setup that maximizes production capacity early on may become less cost-effective during mid-life or may not meet environmental standards in the final stage. Additionally, mine decision-makers should develop a clear transition strategy between phases, ensuring that changes in fleet configuration are made gradually, intentionally, and aligned with evolving priorities. This approach allows for a phased shift from production-focused fleets to economically efficient configurations and ultimately to environmentally sustainable systems, helping to avoid operational shocks, sudden cost increases, and reactive decisions throughout the mine’s life cycle.

Despite the benefits of the proposed framework, its limitations must be recognized. This study concentrates on a specific set of criteria–capacity, capital cost, operating cost, and $CO_2$ emissions–and on a limited number of fleet scenarios based on the actual conditions of the case-study mine. As a result, generalizing the findings to other mines with different geological features, operational sizes, or regulatory environments should be approached cautiously. Additionally, certain dynamic factors–such as rapid shifts in energy prices, market uncertainties, and especially changes in cut-off grade throughout the mine’s life, which can directly impact production levels, extraction planning, and decision priorities–were not explicitly modeled here. Nevertheless, the dynamic and network-based structure of the proposed framework offers strong potential for future development, including incorporating cut-off grade variations, safety and social criteria, and scenarios based on uncertainty.

## Conclusion

Selecting loading and haulage fleets in open-pit mining is a key strategic decision in mining engineering that directly impacts operational performance, economic efficiency, and environmental results. As the vital link between extraction, processing, and the supply chain, this choice significantly influences achieving production goals, controlling costs, and meeting environmental standards. However, this decision is inherently time-sensitive because the technical, economic, and environmental conditions of a mine constantly change throughout its life cycle.

A review of the literature shows that many previous studies–even those using advanced multi-criteria decision-making or optimization techniques–have modeled the fleet selection problem in a static way and have ignored how priorities evolve over the mine’s life. This static view causes an aggregation bias, where a decision that seems acceptable overall may not be optimal during critical project phases. This gap emphasizes the need for a dynamic and time-aware decision-making framework.

To overcome this limitation, this study proposes a dynamic decision-making framework that combines DMCDM with the ANP. By dividing the mine’s life cycle into three key stages–early, mid-life, and final–and establishing a TML baseline, the framework enables phase-by-phase analysis and consistent comparison of fleet options. ANP allows for the explicit inclusion of interdependencies and feedback among technical, economic, and environmental criteria, while phase-specific weighting ensures that criterion importance matches each stage’s conditions.

The results show that priorities among criteria–and therefore the optimal fleet setup–change throughout the mine’s life cycle, and relying on a single, fixed fleet throughout the project may lead to suboptimal choices. Accordingly, this framework can serve as a practical decision-support tool for planning fleet selection, renewal, and replacement in open-pit mines, helping decision-makers align their strategies with the mine’s transition from one stage to the next. Despite these contributions, the study is limited to a specific set of criteria and a finite number of scenarios based on the conditions of the case-study mine. Future research could expand this framework by including additional criteria such as safety, social factors, economic uncertainties, and variations in cut-off grade over the mine’s life cycle. Such extensions would increase the framework’s applicability and improve its ability to support decision-making under more complex and uncertain conditions.

## Limitations and future research directions

Despite the advantages of the proposed methodology and the insights obtained from this study, several limitations should be acknowledged, which also open avenues for future research. First, the set of criteria considered in this study was limited to economic, technical (capacity), and environmental ($CO_2$ emissions) factors. Although criteria such as safety, reliability, and social aspects (e.g., safety, reliability, social aspects) are frequently highlighted in the literature as important considerations in mining equipment selection, they were not included in the present analysis due to the unavailability of reliable and consistent data for the case study examined. Future studies are therefore encouraged to incorporate these criteria, provided that adequate data are available, in order to achieve a more comprehensive and holistic evaluation of haulage fleet alternatives.

In addition, while the proposed framework is based on a DMCDM approach integrated with the ANP, further validation of the results is recommended by applying alternative MCDM methods. Employing other techniques–such as TOPSIS, VIKOR, or fuzzy-based MCDM approaches–would enable comparative analysis of the outcomes and help assess the sensitivity of the rankings to the choice of decision-making method. Such cross-method comparisons would contribute to strengthening the robustness, reliability, and generalizability of the proposed framework and provide deeper insights into potential differences in decision outcomes under alternative analytical perspectives.

## Data Availability

The datasets generated and/or analyzed during the current study are available upon reasonable request from the corresponding author.

## Declarations

All methods were conducted in accordance with relevant guidelines and regulations.

## References

[1] Ghaziania, H. H., Monjezi, M., Mousavi, A., Dehghani, H. & Bakhtavar, E. Design of loading and transportation fleet in open-pit mines using simulation approach and metaheuristic algorithms. *J. Min. Environ.* **12**, 1177–1188 (2021).

[2] Aghajari, A. M. & Namin, F. S. U-HRMES: Decision theory-based model for appropriate mining equipment selection in underground hard rock stopes. *Expert Syst. Appl.* **246**, 123108 (2024).

[3] Rao, R. V. & Davim, J. P. A decision-making framework model for material selection using a combined multiple attribute decision-making method. *Int. J. Adv. Manuf. Technol.* **35**, 751–760 (2008).

[4] Lee, H., Lee, S. & Park, Y. Selection of technology acquisition mode using the analytic network process. *Math. Comput. Model.* **49**, 1274–1282 (2009).

[5] Saleh, N., Gaber, M. N., Eldosoky, M. A. & Soliman, A. M. Vendor evaluation platform for acquisition of medical equipment based on multi-criteria decision-making approach. *Sci. Rep.* **13**, 12746 (2023). 37550351 10.1038/s41598-023-38902-3 PMC10406946

[6] Nguyen, L. H. & Nguyen, T. A. Hybrid mcdm for repair strategy prioritization of port handling equipment: A fuzzy-anp-topsis approach. *Int. J. Adv. Sci., Eng.& Inform. Technol.* **15** (2025).

[7] Saleh, N., Gamal, O., Eldosoky, M. A. & Shaaban, A. R. An integrative approach to medical laboratory equipment risk management. *Sci. Rep.* **14**, 4045 (2024). 38374369 10.1038/s41598-024-54334-z PMC10876531

[8] Rakhmangulov, A., Burmistrov, K. & Osintsev, N. Multi-criteria system’s design methodology for selecting open pits dump trucks. *Sustainability* **16**, 863 (2024).

[9] Avramova, T., Peneva, T. & Ivanov, A. Overview of existing multi-criteria decision-making (MCDM) methods used in industrial environments. *Technologies* **13**, 444 (2025).

[10] Moktadir, M. A., Paul, S. K., Bai, C. & Santibanez Gonzalez, E. D. The current and future states of MCDM methods in sustainable supply chain risk assessment. *Environ. Dev. Sustain.* **27**, 7435–7480 (2025).

[11] Basçetin, A. An application of the analytic hierarchy process in equipment selection at Orhaneli open pit coal mine. *Min. Technol.* **113**, 192–199 (2004).

[12] Bazzazi, A. A., Osanloo, M. & Karimi, B. Optimal open pit mining equipment selection using fuzzy multiple attribute decision making approach. *Arch. Min. Sci.* **54**, 301–320 (2009).

[13] Aghajani Bazzazi, A., Osanloo, M. & Karimi, B. A new fuzzy multi criteria decision making model for open pit mines equipment selection. *Asia-Pacific J. Oper. Res.* **28**, 279–300 (2011).

[14] Campanella, G. & Ribeiro, R. A. A framework for dynamic multiple-criteria decision making. *Decis. Supp. Syst.* **52**, 52–60 (2011).

[15] Subtil, R. F., Silva, D. M. & Alves, J. C. A practical approach to truck dispatch for open pit mines. In *35Th APCOM symposium*, 24–30 (2011).

[16] May, M. A. *Applications of queuing theory for open-pit truck/shovel haulage systems*. Ph.D. thesis, Virginia Tech (2013).

[17] Rahimi Ghazikalayeh, A., Amirafshari, M., Mkrchyan, H. & Taji, M. Application of fuzzy hybrid analytic network process in equipment selection of open-pit metal mines. *Int. J. Res. Ind. Eng.* **2**, 35–46 (2013).

[18] de Sousa Junior, W. T., Souza, M. J. F., Cabral, I. E. & Diniz, M. E. Multi-criteria decision aid methodology applied to highway truck selection at a mining company. *Rem: Revista Escola de Minas* **67**, 285–290 (2014).

[19] Salama, A. et al. Prediction of haul units’ requirements for the open pit mines operating towards closure. *Tanzania J. Eng. Technol.* **38**, 217–229 (2019).

[20] Benlaajili, S. *et al.* Optimization of truck-shovel allocation problem in open-pit mines. In *International Conference on Smart Applications and Data Analysis*, 243–255 (Springer, 2020).

[21] Patyk, M., Bodziony, P. & Krysa, Z. A multiple criteria decision making method to weight the sustainability criteria of equipment selection for surface mining. *Energies* **14**, 3066 (2021).

[22] Mohtasham, M., Mirzaei-Nasirabad, H., Askari-Nasab, H. & Alizadeh, B. Truck fleet size selection in open-pit mines based on the match factor using a MINLP model. *Min. Technol.* **130**, 159–175 (2021).

[23] Huerta, J. R., Silva, R. S., De Tomi, G. & da Silva, A. L. M. A. A dynamic simulation approach to support operational decision-making in underground mining. *Simul. Model. Pract. Theory* **115**, 102458 (2022).

[24] Musbah, H., Ali, G., Aly, H. H. & Little, T. A. Energy management using multi-criteria decision making and machine learning classification algorithms for intelligent system. *Electric Power Syst. Res.* **203**, 107645 (2022).

[25] Mohtasham, M., Mirzaei-Nasirabad, H., Askari-Nasab, H. & Alizadeh, B. Multi-stage optimization framework for the real-time truck decision problem in open-pit mines: A case study on Sungun copper mine. *Int. J. Min. Reclam. Environ.* **36**, 461–491 (2022).

[26] Anaraki, M. G. & Afrapoli, A. M. Sustainable open pit fleet management system: integrating economic and environmental objectives into truck allocation. *Min. Technol.* **132**, 153–163 (2023).

[27] Ccatamayo-Barrios, J.-H. *et al.* Comparative analysis of AHP and TOPSIS multi-criteria decision-making methods for mining method selection. *Math. Model. Eng. Problems* **10** (2023).

[28] Francis, A. & Thomas, A. System dynamics modelling coupled with multi-criteria decision-making (MCDM) for sustainability-related policy analysis and decision-making in the built environment. *Smart Sustain. Built Environ.* **12**, 534–564 (2023).

[29] Namin, F. S., Ghasemzadeh, H. & Aghajari, A. M. A comprehensive approach to selecting mine transportation system using AHP and fuzzy-TOPSIS. *Decis. Making Anal.* 23–39 (2023).

[30] Moradi Afrapoli, A., Upadhyay, S. P. & Askari-Nasab, H. A nested multiple-objective optimization algorithm for managing production fleets in surface mines. *Eng. Optim.* **56**, 378–391 (2024).

[31] Čelebić, M. et al. Development of an integrated model for open-pit-mine discontinuous haulage system optimization. *Sustainability* **16**, 3156 (2024).

[32] Dimitrijević, B. et al. A novel hybrid fuzzy multiple-criteria decision-making model for the selection of the most suitable land reclamation variant at open-pit coal mines. *Sustainability* **16**, 4424 (2024).

[33] Samimi Namin, F. & Soleimani, M. Selection of trolley-assisted hauling systems for open pit mining using grey theory as a green technology. *Green Technol.* **2**, 9–16 (2024).

[34] Amou, A., Ataee-pour, M. & Mahdevari, S. Selecting the optimal scenario for the simultaneous application of diamond wire cutting and chainsaw machines in dimensional stone mines using SECA multi-criteria decision-making method. *Rudarsko-geološko-naftni zbornik* **40**, 83–93 (2025).

[35] Tao, R., Liu, Z., Cai, R. & Cheong, K. H. A dynamic group MCDM model with intuitionistic fuzzy set: Perspective of alternative queuing method. *Inf. Sci.* **555**, 85–103 (2021).

[36] Saaty, T. L. The analytic hierarchy process Mcgraw hill, New York. *Agric. Econ. Rev.* **70**, 10–21236 (1980).

[37] Taherdoost, H. & Madanchian, M. Analytic network process (ANP) method: A comprehensive review of applications, advantages, and limitations. *J. Data Sci. Intell. Syst.* **1**, 12–18 (2023).

[38] Meade, L. & Sarkis, J. Analyzing organizational project alternatives for agile manufacturing processes: an analytical network approach. *Int. J. Prod. Res.* **37**, 241–261 (1999).

[39] Saaty, T. L. Decision making with dependence and feedback: The analytic network process. *RWS Publication* (1996).

[40] Tolvanen, A. et al. Mining in the arctic environment-a review from ecological, socioeconomic and legal perspectives. *J. Environ. Manag.* **233**, 832–844 (2019). 10.1016/j.jenvman.2018.11.124 30600123

[41] Durucan, S., Korre, A. & Munoz-Melendez, G. Mining life cycle modelling: A cradle-to-gate approach to environmental management in the minerals industry. *J. Clean. Prod.* **14**, 1057–1070 (2006).

[42] Hajiabedi, M. M. & Afraei, S. A new mixed-integer linear programming model for ultimate pit limit determination and production planning of open pit mines considering effective operational constraints. *Int. J. Eng.* **39**, 1542–1560, 10.5829/ije.2026.39.07a.02 (2026). https://www.ije.ir/article_226580_18a4d25e7944dc5ad8e80fd4dfd93b79.pdf.

[43] Soon, W., Baliunas, S., Idso, S. B., Kondratyev, K. Y. & Posmentier, E. S. Modeling climatic effects of anthropogenic carbon dioxide emissions: Unknowns and uncertainties. *Climate Res.* **18**, 259–275 (2001).

[44] Shahbakhsh, H., Osanloo, M. *et al.* Greenhouse gas emissions reduction through integration of renewable and non-renewable energy sources: A model for Sangan iron ore mine complex of Iran. *International Journal of Engineering* (2025).

[45] Panahi, S. *Exploratory data analysis and modeling of Gol-E-Gohar No. 3 mine by using of Gemcom software*. Master’s thesis, Amirkabir University of Technology (2014).

[46] Hartman, H. *Introductory Mining Engineering* (John Wiley and Sons Inc., New York, NY, 1986). OSTI record 5757821.

